# Uterine Cervical Neoplasm Diagnosed by Flexible Magnifying Endoscopy with Narrow Band Imaging

**DOI:** 10.3390/diagnostics10110903

**Published:** 2020-11-04

**Authors:** Hideki Kobara, Kunihisa Uchita, Noriya Uedo, Noriko Matsuura, Noriko Nishiyama, Kenji Kanenishi, Tsutomu Masaki

**Affiliations:** 1 Department of Gastroenterology and Neurology, Faculty of Medicine, Kagawa University, 1750-1 Ikenobe, Miki, Kita, Kagawa 761-0793, Japan; n-nori@med.kagawa-u.ac.jp (N.N.); tmasaki@med.kagawa-u.ac.jp (T.M.); 2 Department of Gastroenterology, Kochi Red Cross Hospital, 2-13-51 Sinhonmachi, Kochi 780-8562, Japan; ucchy31@yahoo.co.jp; 3 Department of Gastrointestinal Oncology, Osaka International Cancer Institute, 1-69, Otemae 3-chome, Chuo-ku, Osaka 541-8567, Japan; noriya.uedo@gmail.com (N.U.); ibura9@yahoo.co.jp (N.M.); 4 Department of Gynecology, Faculty of Medicine, Kagawa University, 1750-1 Ikenobe, Miki, Kita, Kagawa 761-0793, Japan; kane@med.kagawa-u.ac.jp

**Keywords:** cervical intraepithelial neoplasia, magnifying endoscopy with narrow-band imaging, endoscopic diagnosis

## Abstract

When detected early, uterine cervical cancer is one of the most successfully treatable forms of cancer. The diagnostic accuracy of the standard method, the Pap smear test followed by colposcopy, remains unsatisfactory. To improve detection of early-stage cervical cancer, new diagnostic tools for uterine cervical intraepithelial neoplasm (CIN) need to be developed. Magnifying endoscopy with narrow- band imaging (ME-NBI), which allows the visualization of the micro-structure as well as micro-vascularity of the mucosal surface, has excellent diagnostic ability for early gastrointestinal neoplasms. In our previous investigation, ME-NBI was efficacious for diagnosis of CIN. We herein report two notable cases of CIN3 that were diagnosed by ME-NBI that were not detected by colposcopy. These cases illustrate the usefulness of ME-NBI for diagnosis of early-stage uterine cervical neoplasms.

## Figures

**Figure 1:**
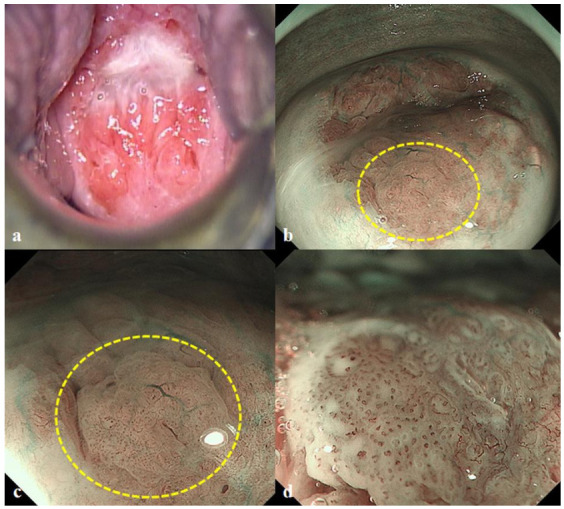
A 39-year-old woman presented with an abnormal Pap smear test result. The initial colposcopy did not reveal any abnormal findings with acetowhite changes (**a**). Then, the patient was referred to our endoscopy unit and underwent magnifying endoscopy with narrow-band imaging (ME-NBI) examination with a gastroscope (GIF-H290Z, Olympus, Tokyo, Japan) on the left lateral decubitus position. First, the NBI image indicated a suspicious abnormal lesion in the 7 o’clock position (yellow circle) (**b**). Next, ME-NBI revealed the thin white epithelium (approximately 10 mm in size) with atypical vessels (yellow circle) (**c**). Atypical vessel was defined as a micro-vessel that satisfied more than two of the following four findings: dilatation, crawling, irregular arrangement, and caliber change according to the intrapapillary capillary loop (IPCL) classification [1]. Finally, 3% acetic acid application showed no of acetowhite epithelium in that area, whereas ME-NBI with acetic acid revealed the atypical micro-vascular architectures with dilatation, crawling, irregular arrangement, and caliber change (**d**) (Supplementary Video S1). The fundamental principle underlying acetowhite epithelium [2] is described as follows: Nucleoprotein is fixed by the acetic acid and the cells become opaque. In normal epithelium, the cells are relatively transparent, allowing visualization of vessels in the stroma; the pink color is due to this underlying vascularity. When the epithelium becomes opaque, the color changes from pink to white because of the reflected light. The intensity of the white is proportional to the amount of nucleoprotein precipitated and the amount of reflected light. Thus, the superficial vessels of cervical intraepithelial neoplasm (CIN)3 tend to be invisible under white light. Conversely, NBI, which is specific light absorbed by hemoglobin, can enhance the superficial vessels as a black color. When applying acetic acid, the surrounded epithelium become white and the vessels become black. Thus, with the color contrast, the vessels are emphasized by ME-NBI. Histological examinations of punch biopsy samples from that area confirmed that the lesion was CIN3. The patient did not complain of any discomfort. Finally, the patient underwent cervical conization, resulting in curative resection of CIN3.

**Figure 2:**
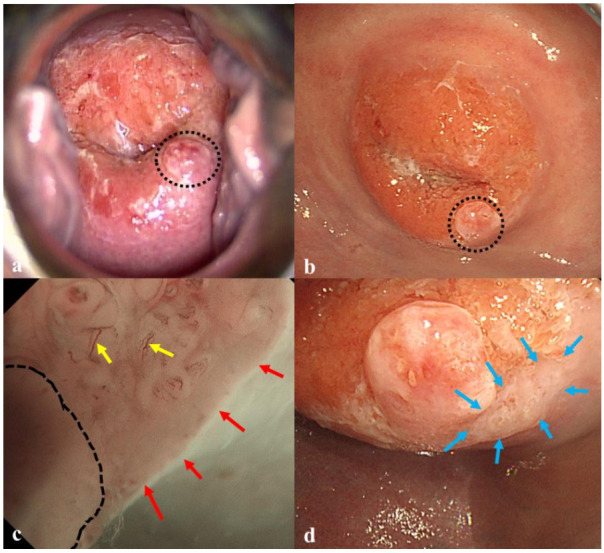
A 37-year-old woman presented with an abnormal Pap smear test result. Colposcopy with acetic acid application showed no acetowhite lesions. An inflammatory polyp was observed in the 5 o’clock position (black circle) (**a**). Then, the patient underwent ME-NBI examination. White light imaging with a gastroscope showed the polyp (black circle) alone (**b**). The ME-NBI revealed a thin white epithelium (approximately 10 mm in size) (red arrows) with atypical vessels (yellow arrows) next to a polyp (black line) (**c**). White light image with acetic acid enhanced the thin epithelium in that area (blue arrows) (**d**). Histological examinations of punch biopsy samples from that area confirmed that the lesion was CIN3. The diagnostic inaccuracy of colposcopy may have been associated with the limited visual field of Cusco’s speculum. Finally, the patient underwent curative resection of CIN3.

The World Health Organization has noted the importance of triple intervention targets to scale up human papilloma virus (HPV) vaccination, screening, and treatment for cervical cancer, and aims to achieve a one-third reduction in the premature mortality by 2030 [3]. The cervical cancer screening program is well-covered by the three test options: the HPV test, the cervical smear, and colposcopy. According to a systematic review [4], the pooled estimates for HPV test sensitivity and specificity were 0.95 (95% confidence interval (CI) 0.84–0.98) and 0.84 (95% CI 0.72–0.91), respectively. The pooled estimates for cervical smear sensitivity and specificity were 0.84 (95% CI 0.76–0.90) and 0.88 (95% CI 0.79–0.93), respectively. In this screening, colposcopy plays an important role in identifying CIN grade two or worse (CIN2+) in detail. A recent quality-controlled review found moderate sensitivity of colposcopy for CIN2+ of 75.1%, specificity of 71.0%, and a positive predictive value of 72.0% [5]. Therefore, some efforts are required to improve the diagnostic accuracy. Automated visual examination, which is a deep learning algorithm for cervix image selection, has been developed [6]. Magnifying endoscopy with narrow- band imaging (ME-NBI) has excellent diagnostic ability for early squamous cell carcinoma in the esophagus, and the head and neck region [7]. Our previous investigations demonstrated that ME-NBI was efficacious for evaluating the micro-vascular pattern [8] as well as white epithelium [9] of CIN. In the CIN appearance, the micro-vessels under ME-NBI correspond to punctuation and a mosaic pattern in colposcopy findings. The irregularity of micro-vessels can be evaluated by ME-NBI, and the grade of CIN is expected to be distinguished according to the IPCL classification [8]. White epithelial neoplasm is endoscopically defined as a well-demarcated whitish lesion with a flat elevation associated with the thickness of the epithelium. ME-NBI, which is able to approximate and magnify the lesion, enables the detection of a flat and elevated white epithelium and identification of the demarcation line of the neoplasm.

Our previous study found that the diagnostic criteria for CIN3 under ME-NBI were: the presence of a thick or thin white epithelium plus atypical vessels or dense acetowhite epithelium grade 2[9]. Both the present cases met the diagnostic criteria for CIN3 under ME-NBI. In our experience, the characteristic ME-NBI findings of CIN2 remain unclear. Commonly, differentiating CIN2 and CIN3 by colposcopy is often difficult; thus, colposcopy is followed by final histological diagnosis. Therefore, the detection of CIN is clinically the most essential. ME-NBI, which can well-visualize the white epithelium as well as atypical vessels, may be advantageous in the detection of CIN.

In the present cases, ME-NBI provided superior visibility of the whole circumferential transition zones and cervix orifice compared with colposcopy. The visual field of the cervix is one of factors that influences diagnostic accuracy. Cusco’s speculum sometimes shows limited ability for patients with a thick cervix and middle aged-patients with regressed transition zones. The maneuver depends on the performer’s experience and skills. To overcome this issue, we developed a vaginal-occluding balloon, providing an alternative for obtaining a clear cervical visual field. Using the balloon mounted on the endoscope tip, the vaginal orifice is occluded, and the field of view of the cervix and vagina is expanded. This instrument is applied in currently ongoing clinical research, and the efficacy has been verified.

These cases illustrated the usefulness of ME-NBI for diagnosis of early-stage uterine cervical neoplasms, creating a new frontier for advanced gastrointestinal endoscopic imaging. A prospective randomized control trial of colposcopy versus ME-NBI is needed to examine the advantage of ME-NBI.

## Supplementary Materials

The following are available online at https://www.mdpi.com/2075-4418/10/11/903/s1, Supplementary Video S1: ME-NBI for CIN.
